# Improving the Thermal Conductivity by Varying the Filler Geometry of Copper in Thermosets

**DOI:** 10.3390/polym18010075

**Published:** 2025-12-26

**Authors:** Uta Rösel, Dietmar Drummer

**Affiliations:** Institute of Polymer Technology, Friedrich-Alexander-University Erlangen-Nuremberg, 91058 Erlangen, Germany; dietmar.drummer@fau.de

**Keywords:** thermal conductivity, thermosets, impact of geometry, particle size distribution

## Abstract

Thermal management is rising in importance due to the evolving requirements of electronic devices, namely, compactness and performance. Polymers, particularly thermosets, exhibit low thermal conductivity, so that fillers are required to enhance the performance of thermosets and make them suitable for such applications. So far, various factors have been investigated in order to improve the thermal conductivity of thermosets, mainly based on single-filler systems. Given the variation in the geometry of different filler types, suggestions about the influence of geometry have also been made. However, the impact of the geometry of the filler type is rather unknown. Therefore, this paper investigates the use of copper (Cu) as a filler with high thermal conductivity and examines four different geometry types (three sphere types with different sizes, as well as platelets) in terms of their reaching a higher thermal conductivity in an epoxy matrix. Cu platelets showed the highest thermal conductivity values, even though they also exhibited high anisotropy. To understand their material behavior in more detail, a new method of inline viscosity measurement is further evaluated. This method allows consideration of local flow conditions and is therefore more precise than methods based on complex viscosity.

## 1. Introduction

### 1.1. Application Fields and Possible Extensions

As modern electronic devices, such as power electronics and electric motors, become more compact and powerful, efficient thermal management is playing an increasingly critical role in their design. This demand arises from the greater amount of heat generated by larger power devices and/or products capable of higher operating temperatures. One example is the silicon carbide (SiC) chip, the potential operating temperature of which has increased from 90 °C [1] up to 250 °C over the past two decades [2].

To withstand such thermal loads, materials used in items such as electronic components or electric motor encapsulations must exhibit high thermal resistance. As the long-term service temperature is higher in thermosets than in thermoplastics, these applications mainly rely on thermosets, particularly epoxy resins [3].

In terms of applications in housing samples for electronic devices, the materials have to provide both high thermal conductivity and high electrical insulation as well. However, polymers in general have low thermal and electrical conductivity. Therefore, fillers are added to meet the required specifications. Compared to thermoplastics, thermosets have the potential for higher filler loadings due to their distinct viscosity behavior and lower viscosity minimums.

### 1.2. Mechanisms of Thermal Conductivity of Thermoset-Based Compounds

Heat transfer in polymers primarily occurs through the propagation of elastic waves along individual macromolecules via covalent bonds. In addition, secondary interactions—such as van der Waals forces—enable heat transfer between adjacent molecules [4]. Intramolecular heat transport, which means heat transport within a molecule, is generally more efficient than intermolecular transport. Thermal conductivity increases with molecular mass, as a higher molecular weight leads to fewer chain ends and, consequently, fewer van der Waals interactions. However, as noted in [5], the increase in thermal conductivity with molecular mass is limited and strongly dependent on the polymer’s chemical structure. Furthermore, an energy balance that includes both conductive and convective components governs heat transfer in polymers. However, most of the heat transfer models, which focus on thermoset-based composites, neglect the convective contribution or do not consider it at all [6]. Thermosets primarily relay on conductive heat transfer after they solidify during curing. With this, no fluid motion is possible, which makes conduction the dominant mechanism. However, during processing and in the curing itself, the resin reveals a low viscosity in the uncured state, so that convective effects may occur. Given this, the temperature distribution can be influenced. Ignoring convection in such cases may lead to inaccurate predictions of thermal behavior and reduced control over composite quality.

Filler systems are essential for enhancing the thermal conductivity of polymers. For example, the thermal conductivity of pure epoxy resin is only 0.4 to 0.6 W∙m^−1^∙K^−1^ [3]. The thermal conductivity of filled epoxy resins can reach values between 10 and 20 W·m^−1^·K^−1^. In [7], a thermal conductivity of 10.98 W·m^−1^·K^−1^ was achieved using an aluminum nitride filler system with a filler loading of 60 vol.-% and a silane surface modification within an epoxy matrix. By combining various anisotropic boron nitride particles, ref. [8] reported a thermal conductivity of 19.0 W·m^−1^·K^−1^, again based on an epoxy resin. To further improve the thermal conductivity of filled thermosets, interfacial thermal resistance (ITR) must be considered, as it impedes heat transfer. ITR can be divided into two types: matrix–filler interfaces and filler–filler interfaces. Minimizing ITR is crucial for enhancing thermal conductivity [9]. Several strategies have been proposed to reduce ITR, including the in situ growth of hetero-structured fillers [10], the surface functionalization of fillers [11], and the alignment [12] or the bridging [13] of fillers. The in situ approach is particularly effective in reducing resistance at filler–filler interfaces, but not in terms of matrix–filler interfaces [9]. However, filler alignment may not be suitable for certain applications, as noted in [14].

Additionally, the intrinsic thermal conductivity of an epoxy resin can be improved by introducing a liquid-crystal structure, which promotes molecular orientation and rigidity on a molecular level [15]. This aspect, however, is beyond the scope of this manuscript and will not be discussed further.

### 1.3. Influencing Factors on the Thermal Conductivity of Filled Thermosets

To maintain electrical insulation while enhancing thermal conductivity, mineral or ceramic fillers are predominantly used [16], whereas high thermal conductivity is reached within metal-based filler systems, which reveal electrical conductivity as well [3]. In terms of a combination of high thermal conductivity and electrical insulation, boron nitride (BN), aluminum oxide (Al_2_O_3_) and zinc oxide (ZnO) are commonly used. Among these, BN exhibits an initial thermal conductivity that is approximately ten times higher than that of ZnO [17]. Typical filler systems with both high thermal and electrical conductivity are copper (Cu), graphite and carbon. Graphite, or, in general, carbon-based fillers, combine the advantages of high possible thermal conductivity and a lightweight aspect [14]. Cu and graphite exhibit similar values for the initial thermal conductivity relative to BN, but carbon fibers reveal about two to three times higher values [18].

Beyond the intrinsic properties of the fillers, several additional factors influence the thermal conductivity of the resulting compound. As outlined in [17], these include the matrix material, filler particle size, particle size distribution and filler geometry. While the filler grade is a key determinant of the thermal performance, the number of thermal contact points also plays a critical role. According to [19], composites with a higher filler loading and a greater aspect ratio exhibit significantly improved thermal conductivity compared to those with lower aspect ratios. This enhancement is attributed to the formation of longer and more continuous conduction paths [20]. Platelet- or rod-shaped fillers generally provide higher thermal conductivity than spherical fillers [4]. These two-dimensional (2D) geometries (platelets, sheets and flakes) offer a large contact area at the filler–filler interfaces, which can result in high interfacial thermal resistance (ITR). However, due to the reduced interstitial volume between particles, the overall ITR may decrease, ultimately leading to improved thermal conductivity [20]. According to [21], the filler shape has a greater influence on thermal conductivity than filler size, particularly for nanoscale particles. Nonetheless, such anisotropic geometries often result in directional heat conduction—i.e., thermal conductivity is significantly higher in one direction, such as along the fiber axis. Moreover, smooth-surfaced fillers are preferred to improve processability and reduce interparticle friction during compounding and injection molding, for example [20]. With respect to maximizing the number of contact points and the size of the contact area, larger particles are generally favored—especially when they exhibit alignment, forming continuous conductive pathways [5]. The positive effect of larger filler particles on thermal conductivity is also linked to their reduced specific surface area, which lowers the total filler–matrix interfacial area and thus reduces ITR [20]. However, ref. [20] also demonstrated that larger particles may negatively impact thermal conductivity due to an increased defect density in the composite. This issue is particularly relevant in graphene-based fillers with excessive oxygen content in the layers [22].

It is important to note that particle size is just one factor influencing thermal conductivity. While larger fillers can increase the number of contact points through direct tangency, they also tend to have a greater average distance between particles than smaller fillers at the same filler loading. When fillers are relatively large, their surfaces are more likely to physically touch each other directly. This direct tangency increases the number of particle–particle contact points, which can enhance the formation of conductive networks within the matrix. However, larger interparticle gaps hinder percolation at low filler loadings. Therefore, using large fillers does not necessarily lead to improved thermal conductivity; other influencing parameters must also be considered.

Hybrid filler systems, which combine both small and large particles, can reduce the interstitial space between fillers and thereby improve thermal conductivity [23]. The nature of the contact between fillers in such systems (whether point or line contact) plays a significant role. Line contacts are preferable, as they provide a larger contact area and facilitate more efficient heat transfer across filler–filler interfaces. However, certain combinations within hybrid systems should be avoided. Specifically, the combination of zero-dimensional (0D) nanoparticles with one-dimensional (1D) structures, such as fibers, rods, tubes, or wires, is not effective in enhancing thermal conductivity [20].

### 1.4. Aim of the Paper

The evolving performance demands in power electronics call for enhanced thermal conductivity in thermoset-based systems in some applications, in addition to electrical insulation. Several influencing factors have been defined with respect to increasing the thermal conductivity of thermosets. Besides the filler grade, the filler shape and size are stated to be two of the most important factors in terms of reaching a thermal flow path, reducing the interfacial thermal resistance (ITR) and with that improving the thermal conductivity. However, most of the investigations so far have compared different filler materials, which also reveal different filler geometries. As the different material systems do not only differ in terms of geometry but also in terms of material-specific properties, such as surface tension, the transfer of the impact of the geometry onto the thermal conductivity reveals inaccuracy if different material systems are investigated. Therefore, this paper examines the impact of the geometry and size for one material type of filler. Copper was chosen as the filler material, as it possesses high thermal conductivity, which is demanded by potential applications in electronic devices, and it is available with a broad variation of geometries. The filler grade was kept constant at 50 vol.-% throughout the investigation; furthermore, an epoxy resin was set as the matrix material. In addition to the changing geometry and size type of the filler, a new approach in terms of analyzing the viscosity behavior was realized. By implementing a dielectric sensor in the injection molding, the ion viscosity was defined throughout the fabrication of the samples. In addition, the ion viscosity was compared with the complex viscosity to evaluate the reliability of the new analysis method.

## 2. Materials and Methods

### 2.1. Materials

The matrix material used in the experiments was an epoxy resin of the type Epoxidur EP 368/1 (Raschig GmbH, Ludwigshafen, Germany), which is a pre-mixture of resin, hardener, catalyst and carbon black pigments, the exact formulation being confidential.

Four different filler types were analyzed based on the material copper (Cu) (Schlenk Metallic Pigments GmbH, Roth, Germany). Three types were based on sphere geometry, namely, Rogal-type copper GK 0|50 (large), 50|100 (medium) and 0|250 (small), and a platelet geometry of the type cubrotec 5000 was also investigated. The filler grade was set at a constant level of 50 vol.-%.

Table 1 summarizes the specification of the matrix and filler material in terms of the density (ρ), the particle size in terms of numerical and volumetrical counting, the heat capacity (c) (based on our own measurements, further discussed in Section 2.3.1) and the thermal conductivity (λ) (based on the manufacturer specifications). In terms of the platelet filler type, the thickness of the particles was between 1 and 2 µm.

**Figure 1:**
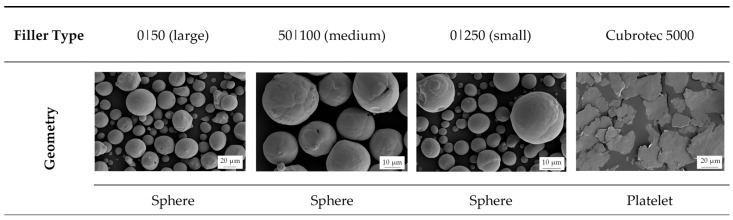
SEM images of the different filler types based on copper (Cu).

In addition, Figure 1 depicts the geometry of the fillers based on images taken by a scanning electron microscope (SEM) of the type Gemini Ultra-Plus (Carl Zeiss AG, Oberkochen, Germany).

### 2.2. Fabrication of the Test Specimens

The fabrication of the test specimens was divided into two steps. In the first step, the compound was produced using a twin-screw extruder (type: Kraus Maffei Berstorff ZSE 25Ax45D; KrausMaffei Group, Munich, Germany) with a constant rotational speed of the screw of 80 min^−1^ and a temperature range from 50 °C to 90 °C from the feeding zone to the nozzle. Thereafter, the matrix and the filler material were pre-mixed manually at room temperature by ensuring the exact amount of each ingredient using a high-precision weighing device. This mixture was compounded on the twin-screw extruder.

**Figure 2:**
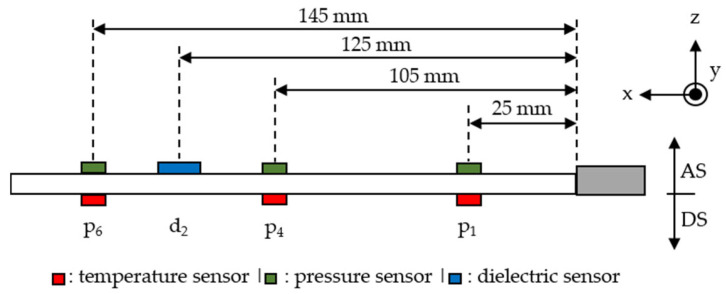
Schematic of sensor positions in the mold of the test specimen during injection molding [pressure sensor, temperature sensor and dielectric sensor] [AS: gate side; DS: nozzle side].

In the second step, an injection molding machine (type: KM 80-380 CX DUR/03; Kraus Maffei Group, Munich, Germany) with a screw diameter of 30 mm was used to fabricate test samples. These plate samples had dimensions of 20 × 200 × 0.5 [mm^3^]. The process parameters were kept constant in the different filler systems, as shown in Table 2. The process parameters of the fabricated samples in the injection molding process were determined. Within the mold, three pressure sensors (type: 6163AAE; Fa. Kistler Instruments GmbH, Winterthur, Switzerland) and three temperatures sensors (type: 6192BSP1; Typ K; Fa. Kistler Instruments GmbH, Winterthur, Switzerland) were included in order to monitor the process conditions. Further, a dielectric sensor (type: DEA 28800A76.040-00; Fa. Netzsch-Gerätebau GmbH, Selb, Germany) was included to identify the ion viscosity based on the movability of the load carrier (dipoles). This movement can be detected in the parameter of the loss factor (ε″), which is the reciprocal of the ion viscosity [24]. The monotrode of the dielectric sensor revealed a diameter of 25.4 mm with an active field of 18 mm. Based on pre-frequency testing between 100 mHz and 100 Hz, the measuring frequency was set at 1 Hz. The exact position of the sensors is depicted in the schematic in Figure 2; the positions were kept constant throughout the test fabrication. The flow direction was in the z direction according to Figure 2.

### 2.3. Characterization

#### 2.3.1. Particle Size Distribution

The particle size distribution was defined using an optical camera with static image analysis (Morphology G3s, Malvern Panalytical GmbH, Kassel, Germany). Up to 80,000 particles in a volume of 5 mm^3^ were analyzed.

#### 2.3.2. Differential Scanning Calorimetry (DSC) According to DIN EN ISO 11357

Differential scanning calorimetry (type: DSC 2500; TA instruments, New Castle, DE, USA) was used to analyze the temperature-dependent reaction kinetics of the material under a nitrogen atmosphere according to DIN EN ISO 11357 [25]. This characterization was essential to identify the process conditions for the fabrication of the test samples. About 5 mg of the material system was heated at a rate of 10 K∙min^−1^ in a temperature range of 0 to 250 °C.

#### 2.3.3. Viscosity Behavior Determined Using a Rotational Viscometer According to DIN EN 53019

To compare the characterization of the viscosity behavior based on the ion viscosity and the dielectric sensor measured during the fabrication of the test sample, a rotational viscometer (type: Discovery Hybrid Rheometer 2, TA Instruments, New Castle, DE, USA) based on two aluminum plates with a diameter of 25 mm and a shearing load were used to define the complex viscosity [26]. After the granulate was positioned on the lower plate at a temperature of 90 °C, which ensured sufficient flow in the material, heating up to 170 °C, which is similar to the mold temperature, was realized at a heat rate of 5 K∙min^−1^. The frequency was kept constant at 1 Hz with a strain of 0.01%. After reaching the starting setup at 90 °C, a shell was placed around the sample and the camber was floated with nitrogen.

In addition, the same setting and measurement program was realized, but a disposable dielectric sensor, similar to the one used in the injection molding section, was placed in the middle of the sample and between the two plates of the rotational viscometer. In this way, the transfer between the viscosity behavior based on the ion viscosity and the complex viscosity could be evaluated.

#### 2.3.4. Density (ρ)

Along the flow path, five test parts with dimensions of 10 × 10 × 0.5 [cm^3^] were prepared using tongs to define the density. The dimensions of the test parts were defined by a digital caliper with a resolution of 0.01 mm. Further, the weight was measured by a high-precision weighing device. Afterwards, the density was calculated using the ratio of weight per volume. In addition, the theoretical density was defined based on the density of the pure materials, as depicted in Table 1.

#### 2.3.5. Specific Heat Capacity (c)

The specific heat capacity (c) was measured using the granulate after compounding at 25 °C using a C80 calorimeter (type: 3D-Calvet calorimeter; TA Instruments, New Castle, DE, USA). After the possible application was seen within a small temperature range, no further investigations regarding the temperature dependence of the specific heat capacity (c) were performed in this paper.

#### 2.3.6. Thermal Diffusivity (α) According to DIN EN ISO 22007

The direction-dependent thermal diffusivity (α) was defined in the x and z directions in the middle of the test sample. The x direction depicts the flow direction (in-plane), and the z direction is perpendicular to the flow direction (through-plane). In terms of a possible application, which could be found in the housing of power electronics, the z direction is more essential. A nanoflash device (type: LFA447; Netzsch GmbH, Selb, Germany) was applied at a temperature of 23 °C according to DIN EN ISO 22007 [27].

#### 2.3.7. Thermal Conductivity (λ) According to DIN EN ISO 22007

Since values for thermal conductivity (λ) are more commonly reported in the literature, this property was calculated based on the thermal diffusivity (α) (in Section 2.3.6), the density (*ρ*) and the specific heat capacity (c), as a product of α, *ρ* and c. In terms of the density (*ρ*), the ideal values were used to focus on the impact of the optimized process.

#### 2.3.8. Filler Distribution

To analyze the filler distribution along the flow path and the possibility of generating a thermal flow path along the fillers with, ideally, a high number of contact points, small strips were prepared. The strips were taken at the positions of the sensors, as shown in Figure 1, using a water-cooled saw with minimum temperature impact. The strips were analyzed in the xy plane, taking images in the z direction (flow direction). The samples were embedded in cold-curing epoxy resin (type: Epofix; Struers GmbH, Ottensoos, Germany) and ground as well as polished afterwards. Images were taken by a stereo microscope (type: Axio Zoom.V16; Carl Zeiss AG, Oberkochen, Germany) with a magnification of 200×. In terms of the platelet fillers, different sections of orientation were defined. The width of each section relative to the total width was calculated as a proportion of the orientation.

## 3. Results and Discussion

### 3.1. Particle Size Distribution

**Figure 3:**
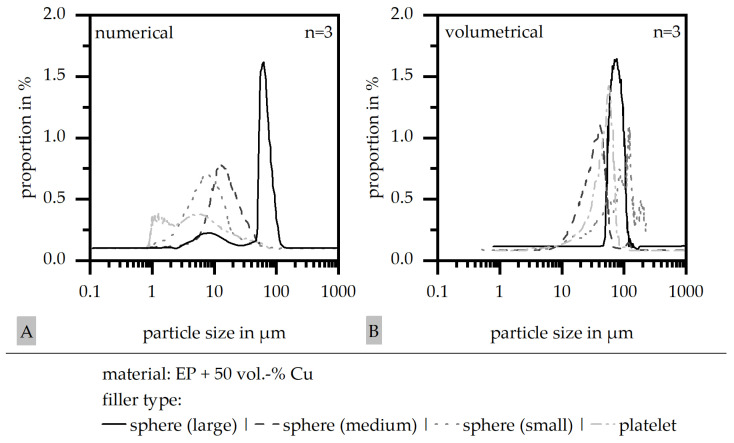
Particle size distribution ((**A**) numerical; (**B**) volumetrical) for four different filler systems (matrix: EP; filler grade: 50 vol.-%).

The particle size distribution in numerical (A) and volumetrical (B) counting is shown in Figure 3 for the four different filler systems. In terms of the numerical distribution, a sharp difference in the particle size between the large spheres and the other fillers can be seen. The platelet filler reveals a broad distribution covering the size range of both the medium- and small-sphere fillers. In the volumetrical counting, the four fillers were revealed to be more similar in their distribution. Again, the large-sphere filler reveals a narrow dispersion, whereas the other three filler types are slightly broader.

### 3.2. Temperature-Dependent Reaction Kinetics Based on Differential Scanning Calorimetry (DSC) According to DIN EN ISO 11357

**Figure 4:**
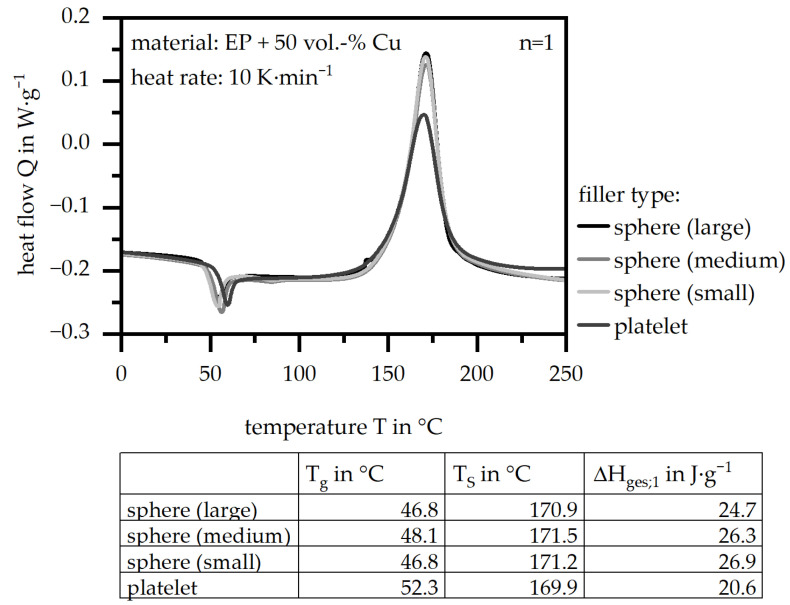
General route of the heat flow (Q) relative to the temperature and three specific parameters (glass transition temperature (T_g_), peak temperature (T_S_) and specific enthalpy (ΔH_ges;1_)) based on DSC measurements for four different filler systems (matrix: EP; filler grade: 50 vol.-%).

Figure 4 depicts the general route of the heat flow (Q) relative to the temperature in terms of the four filler types, as well as the parameters of the glass transition temperature (T_g_), the peak temperature (T_S_) and the specific enthalpy (ΔH_ges;1_). The fillers reveal a similar behavior in terms of the three parameters and the general route, with the exception of the platelet type. This one shows a slightly higher T_g_ and a lower T_S_, but especially a lower ΔH_ges;1_, which results in less required energy to realize the curing process.

### 3.3. Viscosity Behavior

**Figure 5:**
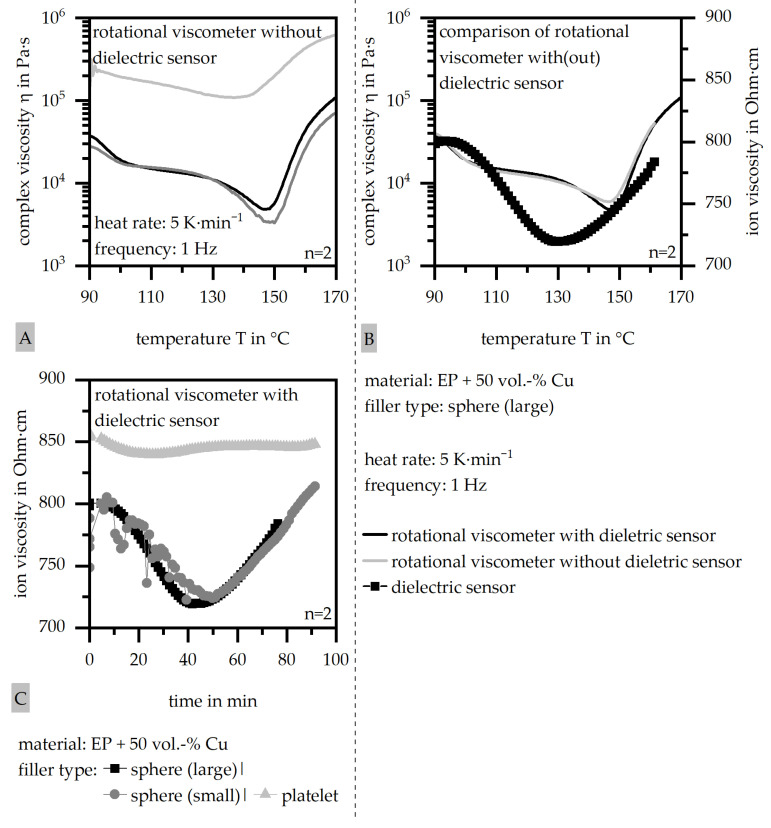
Comparison of the complex viscosity measured by the rotational viscometer with and without a sensor, as well as the ion viscosity, exemplarily for three filler systems (matrix: EP; filler grade: 50 vol.-%) [(**A**) different filler systems measured in the rotational viscometer without the dielectric sensor; (**B**) comparison of rotational viscometer with and without dielectrical sensor and ion viscosity exemplarily for type of sphere filler (large); (**C**) ion viscosity of different filler systems].

To evaluate the viscosity behavior in terms of the measurement in the rotational viscometer and inline during the fabrication of the samples due to the dielectric sensors, Figure 5A depicts the complex viscosity for the large and small spheres, as well as the platelets, characterized by the rotational viscometer without a dielectric sensor. Figure 5B depicts the complex viscosity revealed by the rotational viscometer with and without the dielectric sensor, as well as the ion viscosity exemplarily for the large-sphere filler. In addition, Figure 5C shows the ion viscosity for the three different fillers, similar to Figure 5A.

The complex viscosity is similar for the sphere fillers, independent of filler size. An increase of about one decade can be seen in terms of the platelet filler due to possible building structures, which hinder the flow. These structures, however, may lead to the formation of thermal flow paths, so that the high-viscosity conditions improve the thermal conductivity of the sample due to the flow conditions. Further, the temperature of the minimum of the complex viscosity is reached slightly earlier in terms of the platelets relative to the spheres. It has to be taken into account that the slope of the complex viscosity changes significantly at around 150 °C, whereas the DSC measurement has its peak temperature at around 170 °C. The authors suggest that this shift is explained in terms of the conditions of the measurement and limitations in the accuracy. The complex viscosity was not changed by the application of the dielectric sensor, as shown in Figure 5B. The general slopes with and without the dielectric sensor are parallel, which shows that the dielectric sensors impact the rheology slightly in terms of temperature and time dependence but not general kinetics. Compared to the ion viscosity, the minimum of the complex viscosity increases to about 15 °C later, which equals 3 min in the process. In terms of the ion viscosity, the behavior is similar to the complex viscosity in comparison with the three filler types. Again, the size of the spheres is neglectable, but the ion viscosity increases significantly for the platelets. Again, the minimum is reached earlier for the platelets. With that, the general behavior of the fillers can be characterized by both the rotational viscometer and the dielectric sensor. It has to be taken into account that the dielectric sensor reveals an offset of 15 °C compared to the complex viscosity, which might go along with the global measurement between the plates in the rotational viscometer and the local measurement due to the dielectric sensor. Despite the offset, the authors consider the ion viscosity as another reliable means of characterizing the viscosity behavior of highly filled thermosets. Other than the rotational viscometer, its measurement allows a curing characterization within the fabrication process, as the ionic mobility during the crosslinking process is used as a basis for the inline dielectric sensor. The increase in the electrical-based ion viscosity allows an explicit identification of the curing point.

### 3.4. Monitoring of the Process Conditions

**Figure 6:**
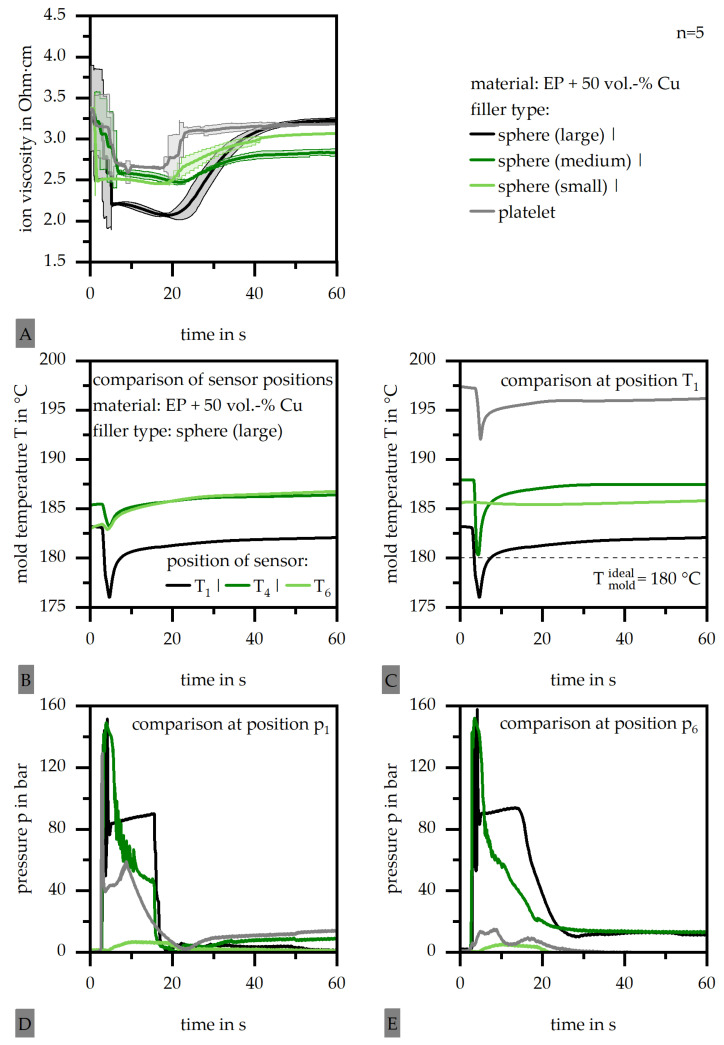
Process conditions for four filler systems (matrix: EP; filler grade: 50 vol.-%) [(**A**) ion viscosity; (**B**) comparison of sensor positions for the mold temperature, exemplarily for one filler type; (**C**) mold temperature at position T_1_; (**D**) pressure at position p_1_; (**E**) pressure at position p_6_].

To understand the material behavior throughout the fabrication and hence the resulting sample properties, the process conditions were monitored, as shown in Figure 6. Figure 6A depicts a comparison of the ion viscosity of the four different filler types. The large-sphere filler exhibits the lowest minimum of ion viscosity but also the longest response behavior in terms of the curing. The platelets reveal the highest minimum of ion viscosity and a fast response behavior. The difference between the complex viscosity (as shown in Figure 5A) of the large-sphere and platelet filler types is similar to the one revealed for ion viscosity. However, the ion viscosity of the medium- and small-sphere ones is similar to that of the platelet ones, which contradicts the results for the complex viscosity. It has to be assumed that the flow conditions within the cavity have an impact on the ion viscosity, so that the level of the minimum ion viscosity is capped. The authors assume a better thermal flow path throughout the smaller spheres and platelet filler type, which results in faster curing and higher levels of the minimum of the ion viscosity. Further, platelet fillers are more likely to hinder the ion mobility relative to spherical ones, which results in general higher specific values.

Figure 6B shows a comparison of the three sensor positions with respect to the mold temperature exemplarily for the filler type of the large sphere. The temperature in the first sensor (position 1) reveals a temperature about 5 °C lower relative to the other two sensor positions. This corresponds to a temperature harmonization along the flow path, where the temperature of the mold and the mass differ more at the gate compared to far away from the gate. In Figure 6C the general mold temperature is compared exemplarily for position 1 for the four filler types. The large-sphere filler type reveals the lowest temperature, with a difference of 15 °C compared to the platelets. This corresponds to the results for ion viscosity shown in Figure 6A, as the higher temperature leads to a faster curing. Further, it has to be taken into account that the initial mold temperature of 180 °C is not met by any of the filler materials. In terms of the platelet type, the actual mold temperature was revealed to be 195 °C, which is significantly outside the setting parameter. The impact of the temperature increase corresponds to the type and geometry of the filler; it is assumed that inner shear between fillers leads to the temperature increase. This shear impact seems to be more relevant in terms of platelet fillers. In Figure 6D,E, the pressure within the mold is compared for the four filler types for positions 1 and 6. The pressure is reduced from the large spheres to the smaller spheres or the platelet type. The smaller the filler size itself is, the greater the pressure reduction revealed along the flow path. This corresponds to the flow conditions, as platelets seem to slide and slip against each other in a more significant way. This corresponds to the higher temperature and the shearing effect, but also results in less required pressure.

### 3.5. Density (ρ)

**Figure 7:**
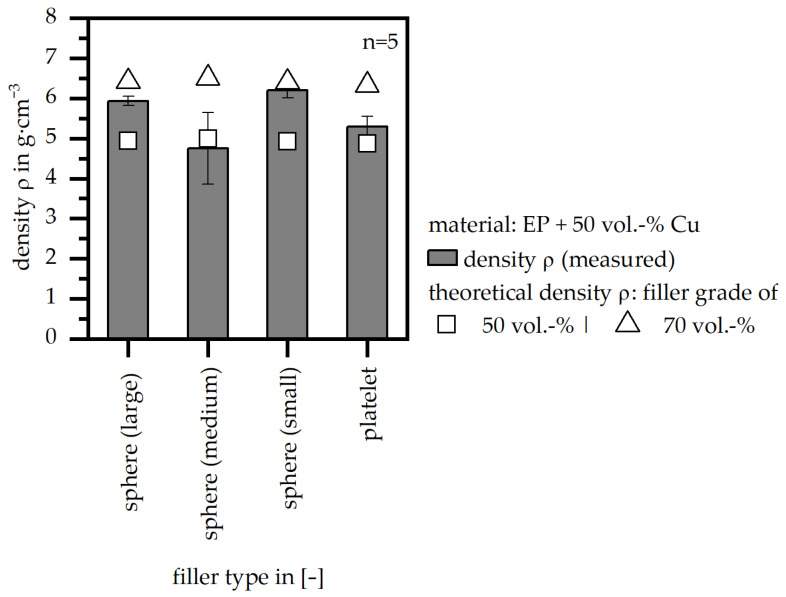
Density (*ρ*) (measured and calculated for 50|70 vol.-% filler grade) relative to four different filler systems (matrix: EP; filler grade: 50 vol.-%).

Figure 7 depicts the measured values of the density (ρ) in comparison to the theoretical values (calculated based on the pure densities of the materials according to Table 1) for the four different filler types. In terms of the theoretical values, two different filler grades of 50 and 70 vol.-% are shown; only the sphere filler type (medium) depicts a lower value out of the measured ones compared to the calculated ones. It can be assumed that the actual filler grade in the test samples and along the flow path differs from the defined one of 50 vol.-%. This leads to different densities along the test sample, which results in broad standard deviations, as well as higher densities, as theoretically calculated. The actual filler grade was further evaluated using a thermogravimetric analysis on the basis of the DIN EN ISO 11358 standard [28] (type: TGA–Q 5000; TA Instruments, New Castle, DE, USA), and the following values were reached: sphere (large): 63 vol.-%; sphere (medium): 46 vol.-%; sphere (small): 67 vol.-%; platelet: 55 vol.-%. In terms of the calculation of the thermal conductivity (λ) in Section 3.6, the theoretical density of a filler grade of 70 vol.-% was applied. Therefore, the proportions of filler and matrix material define the density of the compound.

### 3.6. Specific Heat Capacity (c)

**Figure 8:**
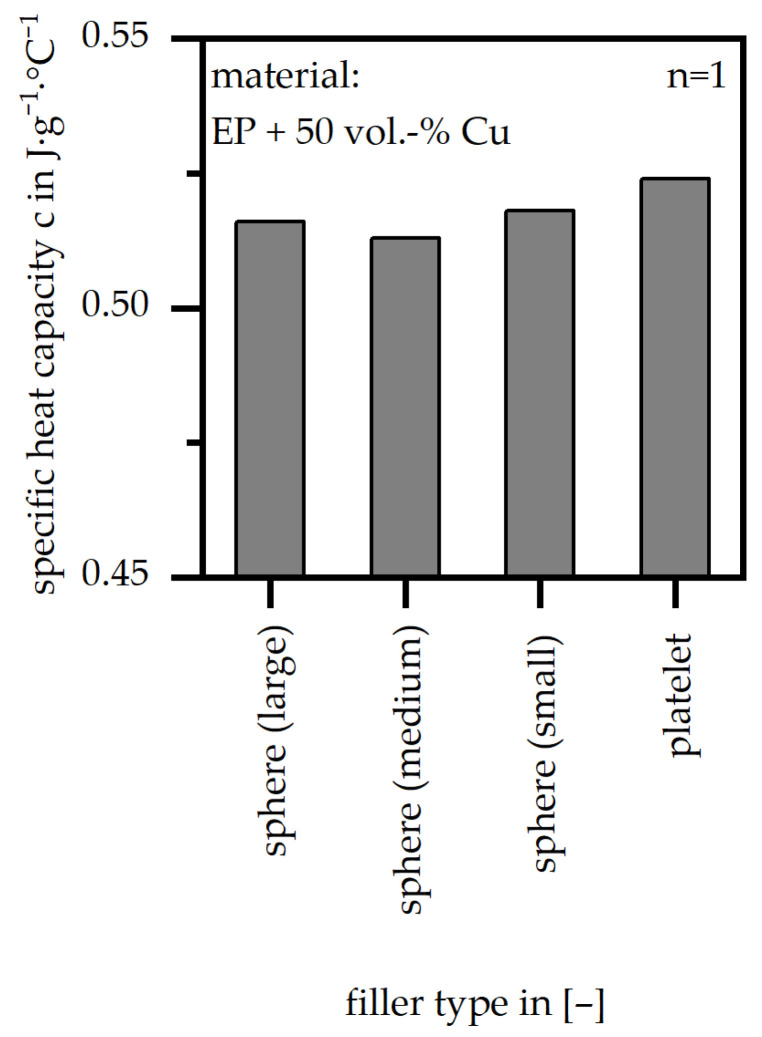
Specific heat capacity (c) relative to four different filler systems (matrix: EP; filler grade: 50 vol.-%).

The difference in specific heat capacity (c) between the four different filler types is shown in Figure 8. With respect to the scale, the difference is very slight, with a tendency to higher values of specific heat capacity (c) for the small spheres and the platelets.

### 3.7. Thermal Conductivity (λ) According to DIN EN ISO 22007

**Figure 9:**
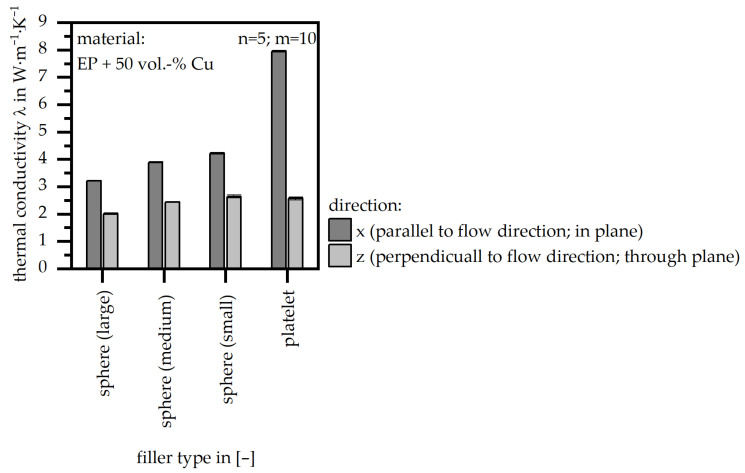
Thermal conductivity (λ) relative to the four different filler types and in two directions (matrix: EP; filler grade: 50 vol.-%).

The thermal conductivity (λ) in- and through-plane is depicted in Figure 9 for the four different filler types. In general, the x direction is about 40% higher compared to the z direction, except for the platelet type, where the difference is more than three times. This corresponds to the geometry, as the platelet structure reveals one direction with a significantly higher thermal conductivity compared to the other. The difference between the sphere-filler size types is slight; however, a smaller size tends to result in a higher thermal conductivity.

### 3.8. Filler Distribution

**Figure 10:**
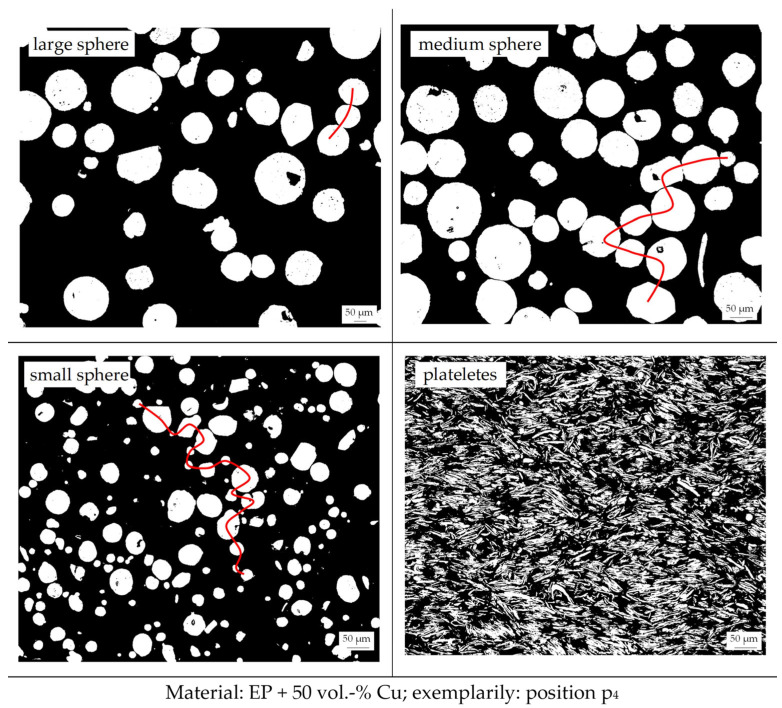
Image of the filler distribution and contact points of the compound material relative to the four different filler types (matrix: EP; filler grade: 50 vol.-%) [exemplarily: position p_4_; red line: possible thermal flow path].

**Figure 11:**
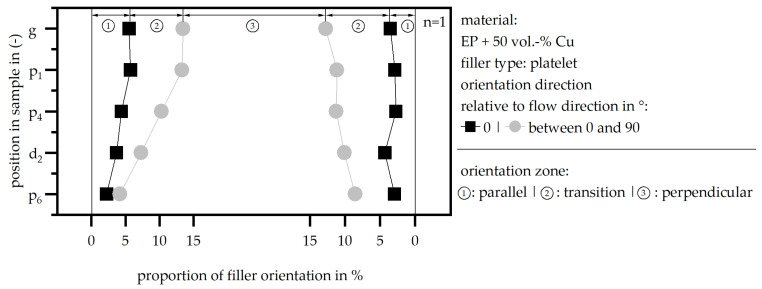
Filler orientation along the flow path and different orientation zones (matrix: EP; filler type: Cu platelet; filler grade: 50 vol.-%).

The filler distribution and the generation of a thermal flow path, as well as the distance between each particle, are shown in Figure 10 exemplarily for the position p_4_. Figure 10 depicts further a possible thermal flow path relative to the different particle sizes of the sphere particles. The differences in the position of the samples, where the microscopy images were taken along the flow path, do not occur for sphere particles. Only for the platelet filler is there a significant difference, as the orientation and the proportion in the cross section differ. In general, the platelets are orientated perpendicular to the flow direction in the middle of the sample. In the edge zone, the orientation shifts to a parallel direction of the fillers relative to the flow direction. This is a likely orientation in terms of thermosets, but this contradicts the state of the literature in terms of thermosets [3]. However, different papers have discussed several other possible filler orientations within thermosets; mainly, the orientation within thermosets depends on the type of filler, according to [29,30,31]. Between the parallel and perpendicular sections of orientation, a transition zone appears, where the fillers are orientated between 0° and 90°. As shown in Figure 11, the proportion of these three areas in terms of the platelets shifts along the flow path.

For the thermal flow path, the smaller spheres reveal the shortest distances between the particles, which corresponds to the higher thermal conductivity, as shown in Figure 9. It is assumed that larger fillers increase the distance between two fillers as the packing density is much lower, as depicted. The large geometry hinders the ability of the particles to reduce the distance between two fillers, so that a significant amount of matrix encapsulates each filler. The smaller the filler, the higher the packing density that can be reached, which leads to more compact thermal flow paths. In terms of the platelets, the distance between each particle is very low; the reduced gap between the particles is more homogeneous along the sample, which leads to a higher thermal conductivity compared to the sphere fillers, especially the small one.

Figure 11 depicts the distribution of the sample in three zones with respect to the filler orientation relative to the flow direction and along the flow path. In general, the fillers are orientated along the flow direction in the edge zone, followed by a transition zone with a random orientation, and in the middle of the sample, the orientation is perpendicular to the flow direction. Along the flow path, the parallel orientation and transition zone are significantly reduced. The authors assume that the parallel orientation is only built near the gate due to the massive change in the cross section. After fast cooling takes place in the edge zones, this orientation cannot be changed afterwards. With each passing material, this parallel orientation is extended with a new layer, which results in a broad parallel orientation near the gate. In terms of the thermal conductivity, the edge zones hinder the increase in the parameter due to the non-supportive orientation of the fillers in terms of the thermal conductivity, especially in the x direction. It can be assumed that an improvement in the flow conditions and a reduction in the different particle orientations could increase the thermal conductivity even further.

## 4. Conclusions

As the shift in performance demands in power electronics requires an increase in the thermal conductivity of thermoset systems, several influencing factors have been identified to enhance the thermal conductivity of thermosets. Besides the filler grade, the shape and size of the filler are among the most important factors for establishing an effective thermal flow path. However, most investigations so far have compared different filler materials which also exhibit different filler geometries. Since material systems differ not only in terms of geometry but also in their material-specific properties, assessment of the impact of geometry on thermal conductivity becomes inaccurate when different material systems are studied in parallel. The authors investigated the same filler material with different geometries in order to evaluate the accuracy of the literature in terms of the impact of geometry on thermal conductivity in thermoset-based systems. It was proven that the impact of the aspect ratio in general is not dependent on the material system of the particle; however, the particle size distribution as an influencing factor relies on the material system of the filler.

Within the manuscript, four different filler types of one material (copper) were analyzed in terms of the flow conditions and the thermal conductivity of samples. Relative to the state of the art, it was proven that higher aspect ratios increase the thermal conductivity after the distance between the particles is highly reduced and better flow conditions can be reached. Further, it was determined that platelets increase the thermal conductivity more significantly than spherical particles do. However, within the manuscript it was proven that smaller particles improve the thermal conductivity of samples due to improved flow conditions, which contradicts the literature. Smaller particles allow the formation of more compact thermal flow paths with a higher packing density.

In addition, the authors revealed a new inline method for defining viscosity behavior. It was proven that the flow conditions of the injection molding had an impact on the viscosity, so that analyses with a rotational viscometer lead to misjudgment, as the real flow conditions in the cavity are not taken into account. In addition, these flow conditions result in different particle orientations in terms of platelets along the flow path.

In addition, a new inline characterization of flow conditions was evaluated and compared with characterization via a rotational viscometer. Further, the flow conditions relative to the geometry of the fillers was examined. It was proven that platelets lead to an inhomogeneous filler orientation along the flow path, mainly because of changes in the cross section from the sprue to the cavity.

Further investigations will be carried out for different filler systems, such as boron nitride, and will examine the variation in the geometry of these materials.

## Figures and Tables

**Table 1:** Specification of the matrix material, including density (ρ), heat capacity (c) (own measurements) and thermal conductivity (λ) (manufacturer specifications).

Material System		Geometry	Density (ρ) in g∙cm^−3^	Particle Size [n|v] in µm	Heat Capacity (c) in J∙g^−1^∙°C^−1^	Thermal Conductivity (λ) in W∙m^−1^∙K^−1^
	Epoxy resin (EP)	-	1.2250	-	1.616	0.4–0.6
Copper (Cu)	0|50 (large)	Sphere	8.65	60.77|76.82	0.351	400
	50|100 (medium)	Sphere	8.77	13.42|33.89	0.360	
	0|250 (small)	Sphere	8.63	6.42|73.31	0.362	
	Cubrotec 5000	Platelet	8.52	5.64|47.38	0.366	

**Table 2:** Process parameters of the fabricated samples in the injection molding process.

Mass Temperature (T_m_) in °C [Feeding|Noozle]	Mold Temperature (T_WZ_) in °C	Heating Time (t_h_) in s	Injection Speed (v_in_) in mm∙s^−1^
65|85	180	75	15

## Data Availability

Restrictions apply for the availability of these data. Data are available with the permission of the author.

## References

[1] Koch I. (2007). Vergleich Si und SiC Halbleiter.

[2] SiC-Halbleiter: Schlüsseltechnologie zur effizienten und Nachhaltigen Elektronik. https://www.siconnex.com/?page_id=6360.

[3] Baur E., Drummer D., Osswald T., Rudolph N. (2022). Saechtling: Kunststoff-Handbuch.

[4] Ehrenstein G.W., Drummer D. (2002). Hochgefüllte Kunststoffe mit Definierten Magnetischen, Thermischen und Elektrischen Eigenschaften.

[5] Übler W. (2004). Erhöhung der Thermischen Leitfähigkeit Elektrisch Isolierender Polymerwerkstoffe. Ph.D. Thesis.

[6] Cai Z. (1990). Simulation of the Manufacture of Closed Shape Composite Structures. Ph.D. Thesis.

[7] Xu Y., Chung D., Mroz C. (2001). Thermally conducting aluminum nitride polymer-matrix composites. Compos. Part A Appl. Sci. Manuf..

[8] Yung K.C., Liem H. (2007). Enhanced thermal conductiity of boron nitride epoxy-matrix composite through multi-modal particle size mixing. J. Appl. Polym. Sci..

[9] Sun Z., Li J., Yu M., Kathaperumal M., Wong C. (2022). A review of the thermal conductivity of silver-epoxy nanocomposites as encapsulation material for packaging applications. Chem. Eng. J..

[10] Fan J., Zhang S., Zhao G., Fu Q. (2021). Constructing fibrillated skeleton with highly aligned boron nirtide nanosheets confined in alumina fiber via electroispinning an dsintering for thermally conductive composite. Compos. Part A Appl. Sci. Manuf..

[11] Shen X., Wang Z., Wu Y., Liu X., Kim J. (2016). Effect of functionalization on thermal conductivies of graphene/epoxy composites. Carbon.

[12] Pan D.A., Yang G., Abo-Dief H.M., Dong J., Su F., Liu C., Li Y., Xu B.B., Murugadoss V., Naik N. (2022). Vertically aligned silicon carbide nanowires/boron nitride cellulose aerogel networks enhanced thermal conductivity and electromagnetic absorbing of epoxy composites. Nano-Micro Lett..

[13] Feng Y., Han G., Wang B., Zhou X., Ma J., Ye Y., Liu C., Xie X. (2020). Multiple synergistic effects of graphene-based hybrid and hexagonal boron nitride in enhancing thermal cundzuctivity and flame retardancy of epoxy. Chem. Eng. J..

[14] Zhou M.-H., Yin G.-Z., Prolongo S.G. (2024). Review of thermal conductivity on epoxy thermosets and composites: Mechanism, parameters, and filler influences. Adv. Ind. Eng. Polym. Res..

[15] Yang X., Zhu J., Yang D., Yang J., Guo Y., Zhong J., Kong J., Gu J. (2020). High-effeciency improvement of thermal conductivity for epoxy omposites from synthesized liquid crystal epoxy followed by doping BN fillers. Compos. B Eng..

[16] Beyer M. (1983). Epoxidharze in der Elektrotechnik: Grundlagen, Verabeitunsganlagen Anwendungen und neue Erkenntnisse.

[17] Johannaber F., Michaeli W. (2004). Handbuch Spritzgießen.

[18] Bard S. (2021). Kohlenstoffaser-Epoxydharz-Verbund mit Erhöhter Wärmeleitfähigkeit: Struktur und Eigenschaften. Ph.D. Thesis.

[19] Zhao Y., Zhai Z., Drummer D. (2018). Thermal conductivity of Aluminosilicate- and Aluminum Oxide-filled thermosets for injection molding: Effect of filler content, filler size and filer geometry. Polymers.

[20] Jasmee S., Omar G., Othaman S.S.C., Masripan N.A., Hamid H.A. (2021). Interface thermal resistance and thermal conductivity of polymer composites at different types, shapes, and sizes of fillers: A review. Polym. Compos..

[21] Liu C., Chen M., Zhou D., Wu D., Yu W. (2017). Effect of filler shape on the thermal conductivity of thermal functional composites. J. Nanomater..

[22] Mahanta N.K., Loos M.R., Zlocozower I.M., Abramson A.R. (2015). Graphite–graphene hybrid filler system for high thermal conductivity of epoxy composites. J. Mater. Res..

[23] Cassing W., Kuntze K., Ross G. (2018). Dauermagnete: Mess- und Magnetisierungstechnik.

[24] Netzsch-Gerätebau GmbH (2025). Dielektrische Analyse. https://analyzing-testing.netzsch.com/de/produkte/dielektrische-analyse-dea.

[25] (2017). Kunststoffe—Dynamische Differenz Thermoanalyse (DSC): Teil 1: Allgemeine Grundlagen.

[26] (2008). Viskosimetrie—Messung von Viskositäten und Fließkurven mit Rotationsviskosimetern—Teil 1: Grundlagen und Messgeometrie.

[27] (2024). Kunststoffe—Bestimmung der Wärmeleitfähigkeit und der Temperaturleitfähigkeit: Teil 1: Allgemeine Grundlagen.

[28] (2022). Kunststoffe—Thermogravimetrie (TG) von Polymeren—Teil 1: Allgemeine Grundsätze.

[29] Schramm G. (1995). Einführung in Rheologie und Rheometrie.

[30] Thienel P., Hoster B., Schröder K., Kretschmer J., Ludwig R. (1993). Duroplast-Spritzgießen mit Gasinnendruck. Kunststoffe.

[31] Englich S. (2015). Strukturbildung bei der Verarbeitung von Glasfasergefüllten Phenolformaldehydharzformmassen. Ph.D. Thesis.

